# Life and Understanding: The Origins of “Understanding” in Self-Organizing Nervous Systems

**DOI:** 10.3389/fnsys.2016.00098

**Published:** 2016-12-09

**Authors:** Yan M. Yufik, Karl Friston

**Affiliations:** ^1^Virtual Structures Research, Inc.Potomac, MD, USA; ^2^Wellcome Trust Centre for Neuroimaging at UCLLondon, UK

**Keywords:** understanding, consciousness, neuronal packets, variational free energy, thermodynamic free energy

## Abstract

This article is motivated by a formulation of biotic self-organization in Friston (2013), where the emergence of “life” in coupled material entities (e.g., macromolecules) was predicated on bounded subsets that maintain a degree of statistical independence from the rest of the network. Boundary elements in such systems constitute a *Markov blanket*; separating the internal states of a system from its surrounding states. In this article, we ask whether Markov blankets operate in the nervous system and underlie the development of intelligence, enabling a progression from the ability to sense the environment to the ability to understand it. Markov blankets have been previously hypothesized to form in neuronal networks as a result of phase transitions that cause network subsets to fold into bounded assemblies, or *packets* (Yufik and Sheridan, 1997; Yufik, 1998a). The ensuing neuronal packets hypothesis builds on the notion of neuronal assemblies (Hebb, 1949, 1980), treating such assemblies as flexible but stable biophysical structures capable of withstanding entropic erosion. In other words, structures that maintain their integrity under changing conditions. In this treatment, neuronal packets give rise to perception of “objects”; i.e., quasi-stable (stimulus bound) feature groupings that are conserved over multiple presentations (e.g., the experience of perceiving “apple” can be interrupted and resumed many times). Monitoring the variations in such groups enables the apprehension of behavior; i.e., attributing to objects the ability to undergo changes without loss of self-identity. Ultimately, “understanding” involves self-directed composition and manipulation of the ensuing “mental models” that are constituted by neuronal packets, whose dynamics capture relationships among objects: that is, dependencies in the behavior of objects under varying conditions. For example, movement is known to involve rotation of population vectors in the motor cortex (Georgopoulos et al., 1988, 1993). The neuronal packet hypothesis associates “understanding” with the ability to detect and generate coordinated rotation of population vectors—in neuronal packets—in associative cortex and other regions in the brain. The ability to coordinate vector representations in this way is assumed to have developed in conjunction with the ability to postpone overt motor expression of implicit movement, thus creating a mechanism for prediction and behavioral optimization via mental modeling that is unique to higher species. This article advances the notion that Markov blankets—necessary for the emergence of life—have been subsequently exploited by evolution and thus ground the ways that living organisms adapt to their environment, culminating in their ability to understand it.

## Introduction

This article offers a synthesis of recent developments in theoretical neurobiology and systems neuroscience that may frame a *theory of understanding*. We suggest that cognitive capacities, in particular understanding, are an emergent property of neuronal systems that possess conditional independencies. In this view, cognition is predicated on associative neuronal groups—or assemblies—that form bounded structures (*neuronal packets*) whose Markov blankets maintain a degree of statistical independence from each other. Such quasi-stable, quasi-independent structures capture regularities in the sensorium, giving rise to the perception of “objects”; namely, the external causes of sensations. These neuronal packets are context-sensitive but maintain their structural integrity. They are composed to form mental (generative) models that reflect the coordinated dynamics of “objects” in the world that cause sensory inputs.

Our basic thesis is that conditional independencies in the causal structure of the world necessarily induce neuronal packets with a similar statistical structure. In effect, the brain “carves nature at its joints” using statistics—to capture the interaction among the factors or causes of sensory data. The implicit factorization of probabilistic representations provides an incredibly efficient process to infer states of the world (and respond adaptively). In physics, this carving into marginal probability distributions (i.e., factors) is known as a *mean field assumption*. Here, we suggest that many aspects of the brain can be understood in terms of a mean field assumption; from the principle of functional segregation, through to the dynamic and context-sensitive maintenance of neuronal packets, groups or cell assemblies. The ensuing theory casts the interaction between the brain and the environment as an allocation of (representational) resources; serving to minimize free energy and thereby maintain homoeostasis (and allostasis).

Variational free energy will figure recurrently in our arguments. Variational free energy is a statistical construct that provides a mathematical bound on surprise or self information (i.e., the improbability of some sensory data, under a generative model of those data). Crucially, free energy is a functional of a (posterior) probability distribution or “belief” about the causes of sensory data—as opposed to a (surprise) function of sensory data *per se*. This means that when a system minimizes its free energy, it is implicitly optimizing its “belief” about the objects that are causing sensory input—based upon an internal or generative model of how that input was caused. Free energy is the difference between accuracy and complexity. This means that minimizing free energy provides an accurate explanation for input that is as simple as possible (where complexity can be construed as a cost function). This complexity reducing aspect of free energy minimization will be important in what follows.

From the point of view of a phenotype, success rests on a deep “understanding” or modeling of the environment. In other words, phenotypes that anticipate and avoid surprising (high free energy) exchanges with their environment possess a generalized form of homoeostasis and implicitly minimize surprise and uncertainty. “Understanding” can therefore be construed as a resolution of surprise and uncertainty about causal structure and relationships in the environment—and in particular the relationship of self to the environment (and others). Differences in adaptive efficiency—between humans and other species—may be determined by formal differences in the generative models used to predict and understand environmental changes over different temporal scales: for example, deep models with hierarchically organized representations vs. shallow models that preclude context-sensitive repertoires of behavior.

This article starts with an overview, followed by four sections: section I reviews theories of understanding in the literature, section II outlines our theoretical proposal, section III presents some empirical findings and examines the correspondence, or absence of such, between our theory and other proposals, section IV re-visits our main suggestions, placing them at the intersection of thermodynamics, information and control theories in systems neuroscience. Our focus in this section is on reconciling the variational (free energy) principles (based upon statistical formulations) with the thermodynamic and homoeostatic imperatives of living organisms—and how these imperatives may furnish a theory of understanding.

### Overview

We pose the following questions:

1. What is “understanding”?
2. What does “understanding” contribute to the overall function performed by the nervous system?
3. What are the underlying mechanisms?
4. How do mechanisms—that can be described in terms of physical processes or information processes (abstracted from physics)—reconcile in a theory of understanding?
5. How does the theory reconcile current views concerning the anatomy and functional architecture of the nervous system?
6. How can one express the theory in a tractable formalism?
7. What is the difference between learning (without understanding) and (learning with) understanding?
8. If the formalism is tractable, what would it entail?
9. What is the key proposal that follows from these considerations?

The article claims no complete answers but suggests where useful answers could be sought. Our framework is system-theoretic, focusing on the general principles of operation in the nervous system. We call on eleven notions: Markov blankets, neuronal packets, self-adaptive optimization, folding, enfolding, unfolding, virtual associative networks, mental modeling, negentropy generation, surface tension and cognitive effort. These and other notions have been elaborated previously (Yufik, 1998a, 2002, 2013; Friston, 2013). For convenience, they are rehearsed briefly in a glossary (please see “Glossary of Terms” below) and will be unpacked as necessary throughout the article.

### Glossary of Terms

*A Markov blanket* is a set of nodes in a network forming an interface between the nodes that are external and internal to the blanket. The conditional dependencies among the nodes endow internal and external nodes a degree of statistical independence within the network: i.e., they are conditionally independent given the states of the nodes in the Markov blanket.

*Neuronal packets* are bounded assemblies (subnetworks) forming spontaneously in associative networks and possessing boundary energy barriers that separate them from their surrounds. Neuronal packets are physical instantiations of Hebbian assemblies, as opposed to information processing abstractions, leading to the conclusion that free energy barriers must exist at the assembly boundary (Yufik, 1998a). This notion predates recent formulations of memory systems as physical devices, as opposed to circuit theory abstractions, and suggests that free energy barriers must exist to “protect” memory states from dissipation (dubbed “stochastic catastrophe”; Di Ventra and Pershin, 2013). Hebbian assemblies devoid of protective energy barriers are subject to “stochastic catastrophe” and dissipate quickly: hence, neuronal packets.

*Self-adaptive resource optimization* is taken to be a principle of operation in the nervous system: the neuronal packet hypothesis views cognitive processes and cognitive development as an optimization of neuronal resources, and considers spontaneous aggregation of neurons into packets as the key mechanism. Thermodynamic energy efficiency is the optimization criteria: the system seeks to maximize extraction of free energy from the environment while minimizing internal energy costs incurred in mobilizing and firing neurons (Yufik, 2002). Resource optimization implies adaptation to changes in the environment as well as to those occurring inside the system (hence, the *self-adaptation*). The notion that spontaneous aggregations (assemblies) of neurons constitute functional units in the nervous system was originated by Hebb, and continues to play a prominent role in theories of neuronal dynamics that focus on the mechanisms of coordination, segregation and integration (e.g., Bressler and Kelso, 2001; Razi and Friston, 2016).

*Folding* denotes the spontaneous formation of regions in networks of interacting units acquiring a degree of statistical independence from their surrounds (i.e., formation of Markov blankets at the boundary). We assume that life emerges in networks that are amenable to folding; thereby regulating material and energy flows across the boundary. This article offers a unifying theoretical framework and explanatory principle for life (and intelligence) that rests on the formation of Markov blankets. The synthesis may reconcile thermodynamic and information-theoretic accounts of intelligence.

*Enfolding* and *unfolding* denote cognitive (deliberate, self-directed) operations on packets: unfolding operates on the internal states of a packet while enfolding treats packets as functional units. Mathematically, enfolding involves computing packet response vectors (the sum of neuronal response vectors), while unfolding reverts to the constituent response vectors. Cognitive processes alternate between enfolding and unfolding; namely, alternating between integrative and focused processing modes. For example, alternations between groups of units (“situations” comprising interacting “objects”) and a focus on particular features of such units (“objects”) and their changes as the situation unfolds. Computationally, the process alternates between matching packet response vector to the input and matching neuronal response vectors. Perceptually, the process manifests, e.g., in grouping visual targets into units, or “virtual objects” and tracking the units, alternating with focusing on and tracking individual targets (Yantis, 1992).

*Virtual associative networks* denote associative networks undergoing self-partitioning (folding) into packets. Mathematically, packets are obtained as minimum-weight cutsets (Luccio-Sami, or LS-cutsets) in networks where nodes are neurons and link weights are determined by the relative frequency of their co-firing (Hebb’s co-firing rule). LS-cutsets “carve out” subsets (packets), such that internal nodes are connected more strongly to each other than to external nodes. In this way, self-partitioning into packets produces a coarse representation of statistical regularities in the environment. Statistically, the nodes of a packet—from which the LS links emanate—constitute its Markov blanket. In other words, they form a boundary, engendering a degree of statistical independence between the packet and its surrounds. Physically, the independence is maintained by energy barriers. The process is similar to structure acquisition in unsupervised learning, except that the quality of learning is adjudicated by thermodynamic constraints. Figuratively, neuronal packets can be viewed as Hebbian assemblies “wrapped” in Markov blankets.

*Mental modeling* denotes self-directed (deliberative, attentive) composition of packets into groups (*mental models*) such that mutual constraints in the packets’ responses can be explored in search of a best fit between implicit models of stimuli. Attaining a good enough fit underlies the experience of reaching, grasp, or understanding. The process improves on and fine-tunes the results of spontaneous packet formation. Mental modeling allows anticipation and simulation of future conditions, and initiating preparations before their onset (anticipatory mobilization of neuronal resources), thus providing a mechanism of neuronal resource optimization.

*Understanding is a form (component) of intelligence. Intelligence* denotes the ability of a living organism to vary its responses to external conditions (stimuli) in a manner that underwrites its survival; e.g., a sunflower following the sun is a manifestation of “plant intelligence” (Trevawas, 2002). Learning is a form of intelligence involving memory and subsequent reproduction of condition-response associations. On the present theory, understanding denotes the ability to compose and manipulate mental models representing persistent stimuli groupings, or “objects”, their behavior under varying conditions, and different forms of behavior coordination (i.e., relations between objects). Understanding overcomes the inertia of prior learning and enables construction of adequate responses under novel and unfamiliar circumstances.

*Negentropy generation* denotes production of information and increases in the order of a system as a result of internal processes. The distribution of weights in associative networks is the result of information intake from the environment (negentropy extraction). Self-directed composition of packets into models increases internal order, without further information intake and without impacting the weights; hence, negentropy generation. Mental modeling amounts to endogenous production of information requiring energy expenditure, the payoff is an increase in adaptive efficiency; i.e., the ability to extract energy from the environment under an expanding range of itinerant conditions. This mechanism enables productive thinking that is sustained by information inflows but is not limited by them.

*Surface tension* is a general thermodynamic parameter defining the thermodynamically favored direction of self-organization in a system. Surface tension corresponds to the amount of free energy in the surface. The neuronal packet hypothesis attributes formation of packets in virtual associative networks to phase transitions (Haken, 1983, 1993; Fuchs et al., 1992; Freeman and Holmes, 2005; Kozma et al., 2005) and accumulation of thermodynamic free energy across boundaries. Boundary free energy barriers are responsible for a packet’s resilience; i.e., the ability to persist as cohesive units—resisting dissipation under fluctuating conditions and entropic erosion.

*Cognitive efficiency* denotes the ratio of free energy extraction (from the environment) and internal energy costs incurred in sustaining energy inflows. The higher the ratio, the higher the efficiency. Mental modeling involves expending free energy to increase internal order (generate negentropy), which entails a more efficient (robust under a wide range of circumstances) energy extraction.

*Cognitive effort* denotes expenditure of thermodynamic free energy incurred in mental modeling. Our theory of understanding associates consciousness with the process—and subjective experience—of exerting cognitive effort. Exerting effort alternates with (relatively) effortless release of genetically supplied and/or experientially acquired (learned) automatisms. Consciousness accompanies the work of suppressing the inertia of prior learning, adjusting learned responses to the current conditions, and composing new responses to anticipate environmental fluctuations. In short, the experience of consciousness is rooted in a high-level mechanism of self-organization and self-adaptive resource optimization in the nervous system. This article focuses on the mechanisms of understanding, postponing a detailed discussion of consciousness for the future.

With these notions in place, the answers to the questions above can be framed as follows:

1. Understanding rests on mental (generative) models representing objects, their behavior and behavioral coordination (i.e., mutual constraints on the behavior of objects).
2. Generative models serve to optimize an organism’s control of its own behavior in a changing environment in the interests of survival (i.e., enduring preservation of structural integrity). The advent of the capacity to understand offered a quantum leap in control efficiency.
3. Control optimization in a changing environment requires anticipatory mobilization of neuronal resources; i.e., progressively improving the ability to select and arrange neuronal representations before the onset of stimuli. Conditioning is the most basic anticipatory mechanism that is shared by all species. The evolution of conditioning to understanding may have proceeded in three stages, predicated on the packet mechanism: Packets capture recurring stimuli groupings. As a result, control efficiency (as compared to conditioning) improved in two ways—by increasing the probability of successful representation and by reducing the cost (i.e., complexity) of internal processing. The formation of packets underlies the perception of *objects*; i.e., bounded stimulus-bound groupings distinct from the sensory background. In the next evolutionary step, the ability to optimize packet allocations (selectively inhibit/amplify neuronal activity within packets) emerged. This ability underlies the apprehension of *behavior*; i.e., changes that objects can sustain without losing their self-identity. Finally, the ability to orchestrate the allocation of packets emerges, giving rise to the apprehension of *relations*; i.e., different forms of behavioral coordination among groups of objects. Apprehending relations requires abstraction from the sensory contents (enfolding): e.g., the relationship of the type “*A rests on B”* defines how the behavior of A coordinates with the behavior of B and vice versa, regardless of how A and B look, smell, sound, etc. Inducing coordinated variations in packet arrangements constitutes *mental modeling*. This capacity supports anticipation into the indefinite future, accounting for large (perhaps, indefinitely large) sets of environmental contingencies.
4. Neuronal firing expends energy. Survival (free energy minimization) is predicated on minimizing the computational cost or complexity of adaptive processing that enables accurate matching of neuronal representations to objects in the environment. In other words, thermodynamic and informational imperatives cannot rely on transitory fluctuations in the system. Instead, a mechanism is needed which produces neuronal structures that withstand entropic erosion and are implicitly available for reuse. It has been suggested previously that neuronal packets are produced by phase transitions in associative networks—and are maintained by “tension” in the surface separating the phases. From an information-theoretic standpoint, *mobilizing* a packet corresponds to inducing a neuronal hypothesis that a particular neuronal packet will provide the best explanation for upcoming sensory input. Accordingly, thermodynamic and information-theoretic approaches converge: the principle of thermodynamic free energy minimization on the packet surface corresponds to the principle of variational free energy minimization in probabilistic inference (Friston et al., 2006; Friston, 2010), both principles referring to the same neuronal mechanism that transcends thermodynamic and variational principles.
5. In what follows, packet variations (selective inhibition/amplification) will be represented as rotation of (population) vectors computed over the internal neuronal states of a packet. On that notion, mental modeling involves the coordinated rotation of packet vectors. For example, motor control is known to entail coordinated rotation of population vectors in the motor cortex. It is not unreasonable to assume that rapid evolution of intelligence in humans expanded the elaborate apparatus of sensorimotor coordination in hominids—to allow packet coordination in the associative cortex and other regions in the brain.
6. The formalism of packet vector coordination for control optimization (self-adaptive allocation of neuronal resources) appears to be tractable.
7. Learning without understanding confines performance to situational envelopes narrowly constrained by past exposures. Understanding expands the envelope indefinitely, enabling counterfactual (“what if”) modeling, simulation of the future—and an implicit ability to “anticipate” the consequences of action.
8. Developing the formalism may help design artifacts to progressively improve their ability to carry out complex tasks, under unfamiliar conditions and unforeseen circumstances.
9. A formal theory appears to be within reach, centered on the notion of Markov blankets, offering a parsimonious account of intelligence that encompasses the transition from inanimate matter to organismal self organization—and from simply sensing the environment to understanding it.

In summarizing, an example may help bring together the perspective on offer: one learns to play chess by first learning to recognize pieces. Learning proceeds by associating different behavioral rules with chess pieces and culminates in the ability to apprehend behavioral constraints (e.g., this black pawn blocks diagonal movement of that white Bishop). Understanding chess involves the ability to apprehend constraints across a composition of pieces—and to determine the possibilities for coordinated maneuvers the composition affords (e.g., “attack on the left flank”). Apprehending behavior coordination requires abstraction (e.g., pin is a form of coordination where the pinned piece shields a more valuable piece behind it). The variety of positions affording this type of coordination is practically infinite. “Chess intuition” collapses its combinatorial space into “lines of play” (Beim, 2012), thus enabling analysis (e.g., 15 moves look-ahead analysis by chess masters (Kasparov, 2007) can be compared to tracing a hair-thin line in combinatorial Pacific Ocean).

## Theories of Understanding

Aristotle’s Metaphysics (350 BC) opens with a statement traditionally translated as “All men by nature desire to know.” Contrary to traditional interpretations, recent analysis (Lear, 1988) suggests that the statement permits a dual interpretation—“to know” and “to understand”; with the latter interpretation being closer to the original intention. Cognition grows out of the capacity to experience puzzlement, accompanied by the feeling of discontentment and desire to resolve it. This capacity to resolve uncertainty is shared by many animals. But only in humans is the desire to resolve uncertainty not fully discharged until a complete understanding is attained (Lear, 1988). Aristotle observed that “animals other than man live by appearances and memories but little of connected experience…” and attributed to men the ability to form connections, i.e., organize disparate data into connected structures. “Wisdom” is attained when such structures reveal causes:

“…men of experience know that the thing is so but do not know the why, while the others know the “why” and the cause”

*—(Metaphysics, book 1)*.

What progress has been made since Aristotle in uncovering the inner workings of understanding? The problem remained largely unaddressed for over two millennia but became prominent in philosophical discourse in the XVIII—XIX centuries (Hume, Spinoza, Berkeley, Kant, Descartes, et al). However, it was not until the middle of the last century that the scope of discourse was radically expanded; largely in response to challenges faced in scientific enquiry, where rapidly accumulating data resisted traditional modes of understanding and explanation (e.g., Bunge, 1979; Cushing, 1994; Sloman, 2005). Philosophy was joined by psychology and cognitive science and, more recently, by what could be defined as *physics of the mind—*an emergent discipline combining statistical physics, information theory and neuroscience to elucidate neuronal underpinnings of cognition (Penrose, 1989, 1994, 1997; Friston et al., 2006; Friston, 2010, 2013). The *physics of mind* framework is consistent with the “enactive” view, deriving cognition from an interplay between external conditions and self-organization in the nervous system. In other words, (non-radical) forms of enactivism enable prediction to guide action on the environment that ensures survival (e.g., Thompson and Varela, 2001). Self-organization places the nervous system in the domain of dissipative systems that are thermodynamically open to the environment. Our proposal for a theory of understanding is thus formulated within *the physics of the mind* framework.

Research areas relevant for understanding include the study of language, consciousness, intentionality, explanation, causality and prediction, logic and reasoning, inference, attention, etc. A detailed review of the relevant research is impossible and is not intended here. What follows is a summary of findings that address some key aspects of the function of “understanding”.

Webster’s Ninth New Collegiate Dictionary defines understanding as comprehension or “mental grasp, the capacity to apprehend general relations of particulars”. This suggests that “understanding” requires a (generative) model that embodies general relationships of particulars; i.e., model that can generate particular consequences from general causes (Craik, 1943; Gentner and Stevens, 1983; Johnson-Laird, 1983, 1989, 2003; Sanford, 1987). Theories of understanding can be roughly organized in five groups, focusing on the different roles of generative models in understanding: (a) volitional (self-directed, deliberate) activity; (b) simulation; (c) need satisfaction and optimization; (d) unification, explanation and prediction; and (e) problem solving. We will reference exemplar theories in each of these groups,—and attempt to relate them to the *physics in the mind* approach.

### Understanding Results from Volitional Operations Targeting Inputs from the Outside and Representations on the Inside

The “foundational theory of understanding” (Newton, 1996) asserts that understanding results from volitional (deliberate, self-guided) actions that involve directing one’s attention to sensory inflows and reconciling current sensations with memory structures in a manner consistent with the current intentions, or goals.

The volitional aspect of cognition is emphasized in the theory of mind-body relationships in Humphrey (2000, 2006). This theory traces volitional activities to their evolutionary origins, as follows. A primitive organism senses physical conditions, or stimuli occurring at its boundary surface and generates commands targeting locations on the surface where the *conditions* were sensed. Commands are said to generate “wiggles” on the surface, the substrates of sensing are not the conditions but the type of “wiggles” produced by the organism adapting to those conditions (e.g., sensing “red” is produced by “wiggling redly, ” sensing “salt” is produced by “wriggling saltily”; i.e., selecting and emitting a response appropriate for the occasion of salt arriving at the surface. Gradually, evolution shifted “response targeting” from surface sites to the efferent, or “sensory nerves” emanating from sites along the surface. Shifting response targets further upstream culminated in the emergence of mechanisms confining responses to internal loops—comprised of efferent and afferent links. In such loops, afferent signals become “as-if commands” (i.e., models): they would have produced appropriate behavior had they been carried all the way to the sensorimotor periphery (Humphrey, 2000, p. 17).

Central to this formulation is the notion of “targeting”; i.e., self-directed mobilization (or recruitment, Shastri, 2001) and focused allocation of neuronal resources. On that notion, an organism is not just registering the flow of sensory impressions but engages in targeted probing and composition of responses fine-tuned to the data returned by sensory samples (consistent with Noe, 2004; Friston et al., 2014). The notion resonates with the sensorimotor contingency, or “action-in-perception” theory (Noe, 2004) and other theories centered on the idea of the “volitional brain” (Libet et al., 2000; Nunez and Freeman, 2014).

Notice the two key themes of this formulation are an emphasis on active inference or volitional sampling of the world—of the sort that characterizes enactivist or situated approaches to cognition. Second, the progressive elaboration of internalized (“as if”) stimulus-response links induces conditional dependencies between the sensory input and internal models of how those predictions were caused—through active sampling.

### Understanding Involves Simulation which is Effortful (Work-Consuming)

Two key characteristics are generally attributed to generative models: models are “structural analogs of the world” (Johnson-Laird, 1983), and models allow simulation of processes and events in the world (Chart, 2000). These characteristics are mutually supportive: if two systems (the world and the model) are formally homologous, one can manipulate and observe the behavior of one system (an internal model) in order to predict and postdict the behavior of the other (an external world). In Chart (2000), simulation is taken to be the essence of understanding, enabling one to both anticipate events and to cope with the unanticipated outcomes. Simulation engages “mutors” i.e., physical mechanisms effecting transformations in the models. The simulation system is hierarchical, including “effectors” responsible for combining “mutors” into groups and attributing meaning and values to the groups, and “simulors” responsible for grouping “effectors.” Crucially, all stages of grouping involve work. An important insight here is that understanding requires the investment of work performed on or by internal representations.

The notion of understanding via simulation can be traced to Craik (1943), who hypothesized the existence of physical mechanisms in the brain functioning as (generative) models of the environment. The theory of understanding in Chart (2000) substantiates this early hypothesis, bringing to the fore a crucial aspect of mental modeling—the necessity to invest work. This was investigated in detail in Kauffman (2000), who postulated that the ability to perform work is the determining factor in perpetuating life and developing capacities that enable an organism to sustain life in a changing environment, while maintaining relative autonomy from it (the emphasis on performing work in the course of mental operations resonates with Freeman et al. (2012) using generalized Carnot cycle to describe process in the cortex). As formulated in Kauffman (2000).

“…an autonomous agent is a self-reproducing system able to perform at least one thermodynamic work cycle…work itself is often used to construct constraints on the release of energy that then constitutes further work. Work constructs constraints, yet constraints on the release of energy are required for work to be done”

*—(Kauffman, 2000, p. 4.)*.

We see here a close connection between (variational) free energy formulations of the imperatives for life that we will return to in the next section. In brief, having a formal physics of mind provides a clear link between understanding (minimization of surprise or variational free energy), a concomitant minimization of thermodynamic free energy and the implicit exchange of work and entropy of a system’s internal representations (by physical states) and the external world to which it is thermodynamically open.

### Understanding Entails Optimization

Generative models improve one’s ability to satisfy homoeostatic needs, when navigating an inconstant and capricious environment—and facing predictable changes as well as the unpredicted (Chart, 2000). Adaptive exchange with the environment is thought of as a measure of need satisfaction (Margenau, 1959; Werbos, 1994, 1998; MacLennan, 1998; Pribram, 1998). Under all circumstances, the activity an agent is engaged in is *the best attempt at the time* to satisfy the current need (hence, the optimization; Glasser, 1984; Werbos, 1998).

The key insight afforded by this perspective is that one can cast all adaptive or intelligent behavior as a process of optimizing some value or need function. In physics, this function is variously known as the *Lyapunov function* or Lagrangian. The existence of this function means that intelligent behavior or understanding can be reduced to “approximate constrained optimization” (Werbos, 1994, p. 40). Again, we see a convergence on optimization or minimization imperatives offered by a physics of mind. In the present context, the objective function is (variational) free energy, where biological imperatives or needs are encoded in prior beliefs about the states a particular agent should occupy. These prior beliefs constrain active sampling of the environment to minimize surprise—and thereby search out preferred states.

Interestingly, the minimization of variational free energy in machine learning is also known as approximate Bayesian inference. In other words, the form of internal modeling that we engage in is quintessentially approximate by virtue of minimizing free energy, as opposed to surprise *per se*. This approximate aspect will become particularly important when we appeal to another ubiquitous device in statistical physics; namely the mean field approximation that provides a clear example of partitioning and functional specialization that may be a crucial aspect of generative models in the brain. We will later suggest that the mental modeling—with mean field approximations in humans—obtains a degree of optimization unavailable to other species.

### Understanding Entails Explanation

According to the Deductive–Nomological (DN) theory of understanding, phenomenon B is understood if particular conditions A are identified along with some appropriate laws such that, given A, the occurrence of phenomenon B is to be expected (Hempel, 1962, 1965). The DN theory was subsequently augmented to account for unification (rendering phenomenon B dependent on phenomenon A must take place in a broader framework, where the number of independent phenomena is reduced), simplification (Kitcher, 1981) or compression (comprehension is compression) and representation of causality (explanation, von Wright, 1971). Establishing causality involves partitioning of A and re-formulating the question “why B?”, as follows:

“Why does this x which is a member of A have the property B?” The answer to such a question consists of a partition of the reference class A into a number of subclasses, all of which are homogeneous with respect to B, along with the probabilities of B within each of these subclasses. In addition, we must say which of the members of the partition contains our particular x”

*—(Salmon, 1970, p. 76)*.

This account of explanation entails an explicit Bayesian formalism (subclasses are hypotheses, encountering B provides evidence) but adds a crucial insight: Explanation is predicated on partitioning heterogeneous A into homogeneous groups, or subclasses. That is, A is a mixed bag, before using the contents for explaining B (and submitting them to Bayesian procedure), they must be sorted into groups that are different (have some features by which they can be told apart) and, at the same time, homogeneous with respect to B. Crucially, partitioning heterogeneous A into homogeneous subclasses is accompanied by production of information and thus requires work. In general, A can admit multiple partitions. Following Carnap (1962), Salmon (1970, 1984, 1989) suggests that the quality of a partition is determined by some utility maximization function imposed at the outset and motivating the investment of work. In this way, Salmon (1970) reveals intimate connections between inference, causality and goal satisfaction.

Establishing causality involves deep inference, or reduction to deeper representation levels (as in seeking the neuronal underpinnings of psychological conditions) as well as determination of intra-level relations (e.g., relating psychological conditions to psychologically traumatic events). Descent to deeper levels in constructing a model (theory) serves to expand the range of surface-level phenomena explained by the model (Dieks and de Regt, 1998). The interplay of the reduction, compression and expansion criteria in constructing models was succinctly defined by Einstein:

“conceptual systems…are bound by the aim to permit the most nearly possible certain (intuitive) and complete co-ordination with the totality of sense-experiences; secondly they aim at greatest possible sparsity of their logically independent elements…”

*—(Einstein, 1949, p. 13)*.

From the perspective of minimizing variational free energy, the implicit many to one mapping between consequences and causes is captured in the notion of minimizing complexity (simplification). Complexity corresponds to the degrees of freedom used to explain data accurately (technically, it is the Kullback-Leibler divergence between a posterior and prior belief). This means that an explanation (to the best inference) is one that maximizes model evidence and minimizes complexity by accounting for a diversity of outcomes (consequences) with the smallest number of plausible explanations (partition of causes).

### Understanding Enables Problem Solving

Arguably, the most extensive and influential body of psychological research on the role of understanding in problem solving was accumulated by Piaget and his school (Piaget, 1950, 1954, 1976, 1977, 1978, Piaget and Inhelder, 1969). Experiments were conducted with young children, which rendered their findings particularly revealing: the problems studied were elementary and their solutions were uncontaminated by prior experience and associations. The main conclusions boil down to the following: problem solving requires establishing relations between “all the multifarious data and successive data” bringing the relations into “*co-instantaneous mental co-ordination*” within a simultaneous whole (i.e., generative model; Piaget, 1978, p. 219).

The notion that problem solving involves “*co-instantaneous co-ordination*” in generative models, thereby imposing simple explanations for “all the multifarious data and successive data” extends from elementary problems solved by children to the highest reaches of theoretical abstraction:

“The general theory of relativity proceeds from the following principle: Natural laws are to be expressed by equations which are co-variant under the group of continuous co-ordinate transformations. …The eminent heuristic significance of the general principles of relativity lies in the fact that it leads to us to the search for those systems of equations which are *in their general covariant* formulation the *simplest ones possible*…”

*—(Einstein, 1949, p. 69)*.

Mathematical equations are expressions of relations between variables; similarly, systems of equations express co-ordination between groups of such relations (Sierpinska, 1994). Accordingly, understanding mathematical formalisms boils down to grasping the relations they entail:

“…if we have a way of knowing what should happen in given circumstances without actually solving the equations, then we “understand” the equation”

*—(Feynman et al., 1964, cited in Dieks and de Regt, 1998, p. 52)*.

Visualization plays a role in problem solving and scientific understanding (van Fraasen, 1980) albeit a limited one. According to self-reports by a number of prominent scientists, the role of verbalization is even less significant (Einstein, 1949; Poincare, 1952; Hadamard, 1954; Penrose, 1989). For example, in his often quoted letter from to Hadamard, Einstein asserts that words hardly participate in his thinking, which consists of “combinatorial play with entities of visual and muscular type…words have to be sought for laboriously only in the secondary stage” (Hadamard, 1954, p. 148). Such self-reports are consistent with experimental findings indicating that verbalization does not facilitate problem solving and can, in fact, interfere with the process (Schooler et al., 1993). They also accord with the analysis of causality placing strong emphasis on the notion that mind establishes causal relations based on mental events, as opposed to verbal accounts that are subsequently formulated (Davidson, 1970, 1993).

### Summary

If not through words and images, then what is the medium of understanding? The perspectives reviewed in this section implicate complexity reduction through factorization and partitioning to explain heterogeneous data. Accordingly, the cardinal aspects of understanding can be formally summarized in terms of minimizing surprise (or free energy) that necessarily entails a generative model of coordination and relations—a model that provides an accurate (unsurprising) and minimally complex explanation for past sensory inputs and predicts forthcoming experiences, including the likely consequences of one’s own actions. We now turn to the mechanisms responsible for such modeling.

## Towards a Theory of Understanding

Following Johnson-Laird (1983), one can distinguish three cognitive mechanisms—symbol processing, image processing and mental modeling: with the latter denoting connected representations and operations on these representations. Our theory is confined to internal modeling, and refers to the process and outcome of such modeling as situational understanding (or *situated cognition*). Cognitive operations underlying the development and exercise of understanding are different from—and do not reduce to—those involved in learning via pattern recognition. The following examples help to appreciate the distinctions.

Fishes can be trained to recognize geometric shapes; e.g., circles (Siebeck et al., 2009). Humans can recognize shapes, name them and, ultimately, define them (e.g., circle is a set of all points in a plane equidistant from the center), which does not yet amount to understanding. A true generative model of a circle comprises representations and operations that enable one to create or manipulate a circle—in practice or “in mind” and at will. For example, the model should account for experiences like handling a circular object, following a circular path, performing circular movements, etc. Having examined a circular object with the eyes closed (e.g., passing a hoop between the palms), one can conjure up an image of a circle; situational understanding manifests, for example, in expecting (not being surprised by) the sensation of a circular edge on palpitating a coin, visually or haptically. These abilities require a generative model; they are distinct from simply recognizing objects or associating symbol strings (names, formulae, descriptions, definitions, etc.) with such objects. In short, understanding is quintessentially enactive and “embodied” (Lakoff, 2003), requiring one to actively engage with the causes of sensations. In the setting of enactive cognition, this means that understanding requires generative models that define affordances for action offered by sensory cues.

**Figure 1:**
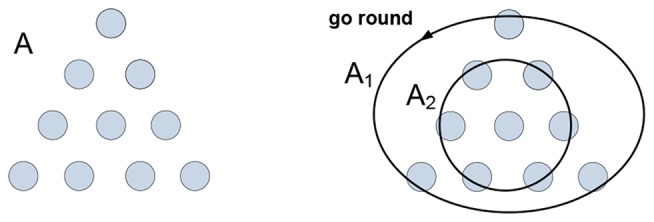
**The arrangement of coins A needs to be inverted in a minimal number of moves.** The solution is obtained by partitioning arrangement A into groups A_1_ and A_2_ allowing their co-instantaneous co-variation: A_1_ “goes round” A_2_. The mental act of “going round” is the medium and the gist of the concept “circle.”

Generative models produce meaning; the meaning of “circle” rests on a model that enables one to do “circling” in the mind (stated differently, the meaning resides in the ability to “wiggle roundly” as the meaning of “red” resides in the ability to “wiggle redly” (Humphrey, 2000)). When fishes are trained to recognize shapes, these shapes acquire significance (predict feeding) but not meaning, fishes form connections but make no sense of them. To appreciate the distinction, note that the definition of “circle” resists visualization (the set of *all* points in a plane equidistant from one point), while the image in your mind is by no means suggestive of the definition. What is then the connection between the definition and the image, what is holding them together? Consider the problem in Figure 1.

Group A_1_ is not a “circle-like” pattern that can be “recognized” in A, nor group A_2_ can be “recognized” as a “point-like” pattern in A, and neither group would be likely to emerge in A had the task been different. Grouping is imputed to A, as opposed to being recognized in—or somehow extracted from—it. The emergence of groups is concomitant with their “co-instantaneous co-variation.” Groups A_1_ and A_2_ are homogeneous with respect to the “go round” variation; the activities of grouping and co-variation in the context of the task yield understanding and determine visualization and verbalization of the solution they produce. To summarize, understanding is yielded by generative models representing objects, behaviors and behavioral constraints. How do such models form and operate in the nervous system?

### Representing Objects

**Figure 2:**
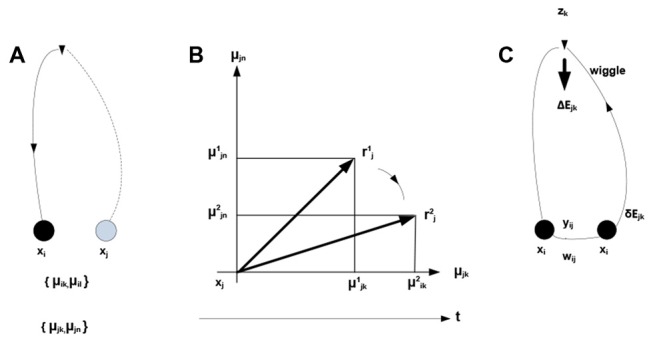
**Development of associative links.** *μ*_ik_denotes probability that neuron *x*_i_ fires in the presence of condition *z_k_*. **(A)** Firing of neuron *x*_i_ initiates mobilization of neuron *x*_j_ having response field overlapping with that of *x*_i_ (both neurons respond to condition *z*_k_). **(B)** Mobilization involves adjustments in *x*_j_—the overlap component (*x*_jk_) is amplified and the non-overlap one (*x*_jn_) inhibited. The adjustment amounts to rotating a neuron’s response vector *r*_j_ (changing from $r_{\text{j}}^{1}$ to $r_{\text{j}}^{2}$). **(C)** Firing of *x*_j_ (sending a “*z*_k_ wiggle”) triggers release of energy Δ*E*_jk_ invested, in part, in producing synaptic modifications establishing an associative link *y*_ij_ of strength *w*_ij_. Mobilization and firing of *x*_j_ consume energy δ*E*_jk_ (other energy expenditures are not represented).

Within the theory of neuronal packets, distinct and bounded entities or objects are recovered from sensory streams as a result of folding in associative networks producing bounded subnetworks (neuronal packets). Associative links form between co-firing neurons, where firing is orchestrated by optimization (free energy minimizing) processes allocating neuronal activity to the stream of stimuli. In this view, free energy is the underlying universal currency in the organism-environment exchange: neuronal firing expends and dissipates energy, while successful neuronal activity extracts energy from the environment. The expending-extracting cycle in the formation of links is illustrated in Figure 2.

**Figure 3:**
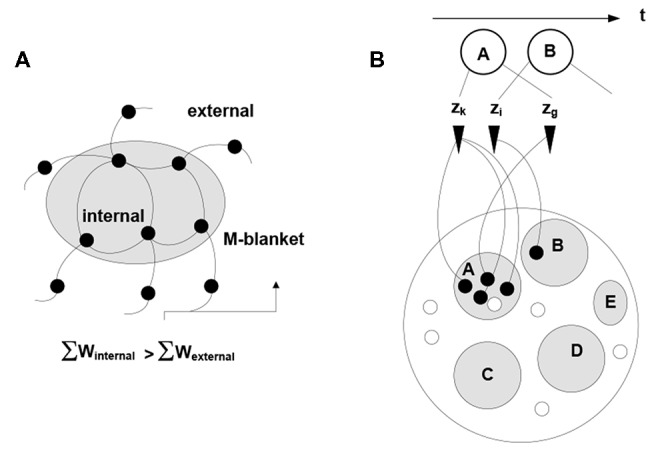
**Induction of Markov blankets and formation of neuronal packets. (A)** Packets are internally cohesive and weakly externally coupled neuronal groups forming in associative networks as a result of a phase transition. Surface tension in the phase-separating surface causes groups to fold into bounded cohesive units capable of withstanding entropic erosion (Yufik, 1998a, 2013). Folding induces Markov blankets at the packet boundaries, that is, makes packets statistically independent (to a degree) from their surrounds. **(B)** The mechanism recovers persistent stimuli groupings A, B,‥.interspersed within the stream; thus giving rise to perception of bounded entities, or objects persisting though (re-emerging in) multiple episodes.

Note the dual nature of the process in Figure 2: on the one hand, the process is a thermodynamic cycle, where energy is received and expended in performing work. On the other hand, mobilizing *x_j_* amounts to forming a hypothesis—entailed by *x_i_*—about the identity of the stimulus, with subsequent validation. The two thermodynamic and information-theoretic perspectives are united by the fact that validation comes in the form of a thermodynamic reward and invalidation entails unrecoverable energy consumption. Associative links decay but are reinforced with every subsequent co-firing of linked neurons. Due to response field overlap, across the neuronal system, a connected associative network gradually forms with the distribution of link weights reflecting statistical regularities in the sensory stream (i.e., repetitive co-occurrence of the stimuli). It has been hypothesized (drawing on the principles of Synergetics (Haken, 1983, 1993)) that the development of the network is punctuated by phase transitions, occurring in tightly coupled subnetworks and causing their folding into bounded aggregations (neuronal packets; Yufik, 1998a,b) Packets are internally cohesive and weakly coupled to (have a degree of statistical independence from) the rest of the network. That is, folding induces Markov blankets in the neuronal pool, as illustrated in Figure 3.

Again, firing of any neuron within a packet mobilizes the entire packet, amounting to the neuronal hypothesis that subsequent stimuli are likely to come in a cluster represented by the neuronal group within the packet. Packet boundaries circumscribe a reference set for the hypothesis, i.e., confine validation probes to the packet internals. Boundary energy barriers discourage but do not prohibit switching reference sets, because unsuccessful probing causes the process to transit to another packet. The packet mechanism is thermodynamically-motivated: energy intakes over time are increased while losses are reduced. If the environment changes, causing diminishing intakes and mounting losses, packets dissolve and are re-constituted.

### Representing Behavior

**Figure 4:**
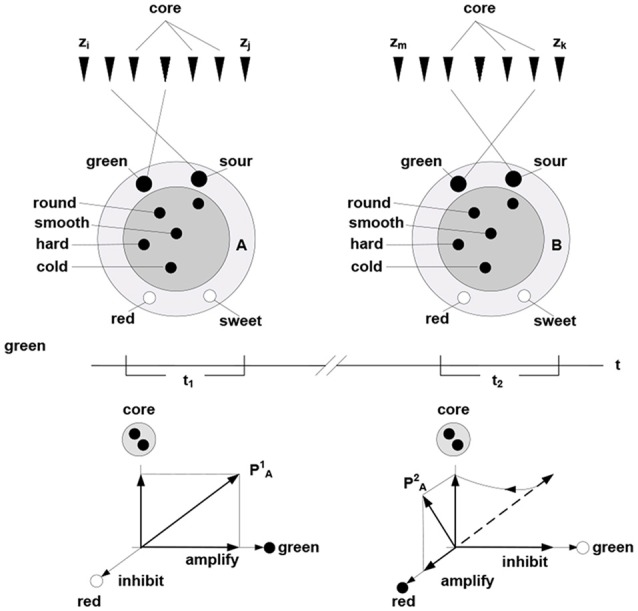
**Packet representation of a round, smooth, hard and cold object changing from green and sour in the time period *t*_1_ to sweet and green in the period *t*_2_ (persisting no longer because the object was eaten).** The subset of neurons in the fringe admits different transition trajectories between the initial and final condition obtained by selective inhibition-amplification of the constituent neurons. The {green, sour} → {red, sweet} transition (behavior) amounts to rotation of packet vector **P_A_** in the hyper plane determined by the fringe subset. Behavior of the object over time is represented by the $P_{\text{A}}^{1}$ to $P_{\text{A}}^{2}$ rotation.

In this formulation, cohesive and bounded neuronal packets act as functional units in the inference process. Stated formally, packet vectors (population vectors) are established on the collectives comprising response vectors of the constituent neurons **P_A_** = (*r*_k_, *r*_h_,…, *r*_g_), here **P_A_** is population vector established on packet A. Allocating packets entails their adaptive adjustments, via selective inhibition and amplification of the constituent responses. The persistence of packets establishes an invariant (slowly varying) core in the setting of a variable periphery, which amounts to formation of a hyperplane in the packet’s response space; thereby confining rotation of the packet vector. Figure 4 illustrates representation of behavior via packet vector rotation (ripening apple changes from green and sour to red and sweet).

The rotation of a packet vector does not violate the object’s self-identity established by the packet or the ability to induce rotation at will, including reversal (e.g., the green and sour object I experienced earlier and the red and sweet object I experience now are one and the same object, which is established, in part, by my ability to revert to the earlier experience and follow its transformation into the present). Reversibility is a determining characteristic of cognitive mechanisms that enables reasoning (no reasoning is possible if, having initiated a thought, one can’t return to the starting point) and apprehending causality (Piaget, 1978).

### Representing Coordination

**Figure 5:**
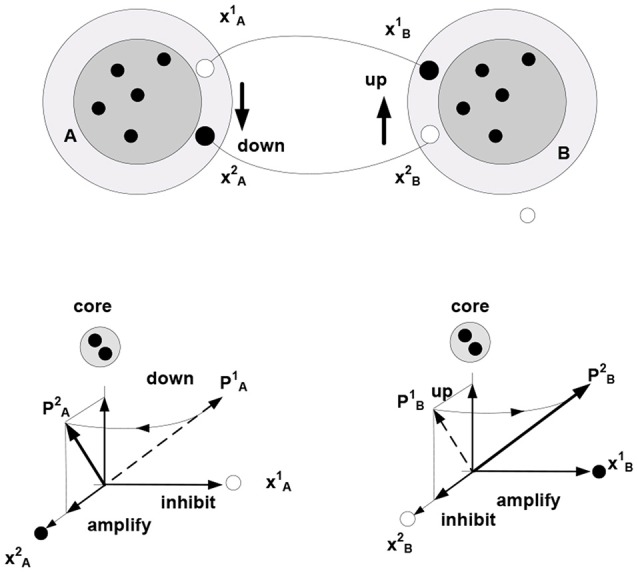
**The behavior of objects A and B (e.g., toy balance) is observed to be coordinated (moving A down is accompanied by B going up, etc.)** Different movements correspond to (are represented by) different successions of firing in the fringes of packets A and B (e.g., neurons $x_{\text{A}}^{1}$ and $x_{\text{A}}^{2}$ represent different positions of A respective the vertical axis, successions $x_{\text{A}}^{1}$ → $x_{\text{A}}^{2}$ and $x_{\text{A}}^{1}$ → $x_{\text{A}}^{2}$ represent downward and upward movement, respectively). Bi-directional mapping between the fringe subsets in two packets establishes “co-instantaneous coordination” in the movement of packet vectors, **P_A_**
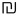
**P_B_** (symbol 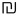 denotes “co-instantaneous coordination” in the movement of packet vectors).

In the present setting, the term “relationship” is taken to denote a form of coordination in the behavior of related objects. Imputing a particular form of coordination to changing (behaving) objects affords a model of the causal dependencies generating sensory data. Establishing coordination in the behavior of objects A and B involves the creation of a bi-directional mapping between the varying subsets (fringe subsets) in the corresponding packets—entailing a coordination of the rotation of packet vectors. Figure 5 illustrates this notion using a task employed in Piaget, to examine development of understanding in young children: discovering how to use a toy catapult (a plank balancing on support) to hit target objects with a plastic ball. Performing the task requires one to understand that pushing down one side causes the other side to go up. That is, “co-instantaneous coordination” needs to be established (Piaget, 1978).

**Figure 6:**
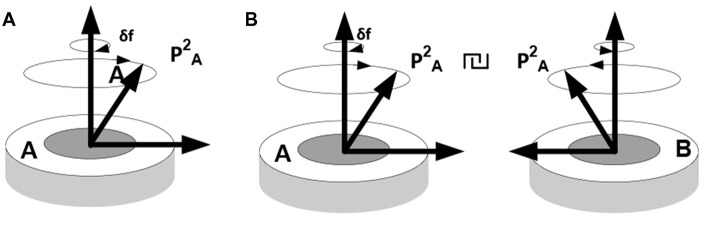
**Establishing behavioral coordination. (A)** Rotation of packet vectors (selective inhibition-amplification of neuronal responses in the fringe subset) is analogous to rotating a shaft in a mechanism, e.g., a dc motor: it requires effort to produce movement (effort δf per unit angular displacement). **(B)** Establishing behavioral coordination creates a unified compositional structure such that vector rotations in one part of the composition produce (enforce) rotations in the other parts (e.g., visualizing one side of a catapult going down brings about, irresistibly, the image of the other side going up, or thinking of holding an object in the hand and releasing the hold brings about the image of a falling object, etc.).

Three important observations are in order here. First, coordinating objects essentially constrains their behavior; i.e., reduces their degrees of freedom or complexity. Establishing coordination between objects in the course of some inference requires representations of the objects and their behavior (situated cognition) but does not reduce to simple recognition. That is, unlike objects and behaviors, coordination cannot be observed but has to be imputed, resulting in a compositional representation (iterative model), such that operations on one part of the composition produce particular changes in the other. For example, when thinking of pushing down one side of a catapult, one cannot help thinking that the other side will go up. The underlying mechanism is neither an image (although some visual predictions might be generated by the model) nor a linguistic expression, such as a rule (although some linguistic predictions might come to mind) but a forceful (energy consuming) mental activity directed at performing a particular work on a representation (vector rotation). Figure 6 illustrates this notion.

In the absence of coordination, packets A and B are experienced as unrelated objects displaying mutually independent behavior patterns. Establishing coordination in the movement of packet vectors produces a generative model; that is, a coherent representative structure (model) and constrained operations on that structure (mental modeling), giving rise to the experience of a unified construct that combines objects in a meaningful relationship.

Figuratively, population vectors can be taken to represent the “consensus view” of the population, while vector rotation expresses changes in neuronal responses in the course of “settling on” a “consensus”. According to the current proposal, understanding involves coordinated neuronal activities (Bressler and Kelso, 2001, 2016), in particular, coordinated rotation of population vectors comprising in a mental model, with the form of such coordination reflecting the form of mutual constraints (dependencies, relations) in the behavior of the entities represented by the populations. Consistent with that proposal, the experience of “grasp” accompanies the concluding stage in the modeling process that “settles” onto a consensus regarding relations among the participating entities. In short, settling onto the “consensus view” in a model corresponds to obtaining mutually coordinated vector rotations across the model representing a coherent account of the situation as it unfolds.

Second, exerting cognitive effort is hypothesized to be a correlate of consciousness (Yufik, 2013). Associative links and their spontaneous groupings (packets) are the product of learning; i.e., they condition the organism to emit recurring responses under recurring circumstances. Effortful composition of packets into mental models and model manipulations (e.g., coordinated rotation of packet vectors) serve to overcome the inertia of prior learning, when encountering and/or anticipating unfamiliar conditions. Learning capabilities are common, to a varying degree, to all animal species, a superior adaptive efficiency in humans may be due to mechanisms allowing effortful suppression of the automatisms acquired in learning and/or adjusting their execution—depending on the circumstances at hand.

Third, coherent neuronal structures are thermodynamically beneficial; i.e., resisting decomposition and/or reorganization. For example, young children fail to understand that, when the target is moved away from the catapult, the ball’s position on the plank needs to be shifted in the opposite direction. Failure is caused by the previously established basic coordination (reaching an object requires movement towards it, not away from it) precluding the requisite adjustments (children are incapable of a focused cognitive effort demanded by the adjustment).

Formally, coordination of packets defines an objective function over a vector space. In the nervous system, the function is implemented in a structure that is analogous (within limits) to Shannon’s Differential Analyzer (DA; Shannon, 1941). The DA machine is composed of shafts connected by movement conveying devices such as gear boxes. When a shaft representing an independent variable is turned, all other shafts are constrained to turn accordingly. The implications of this analogy will be examined elsewhere, excepting the following observations.

(a) The objective function seeks maximization of energy efficiency, that is, vector (shaft) rotations are sought that maximize energy inflows at the expense of minimal rotation effort.
(b) A coherent model (tightly coordinated packets) collapses combinatorial complexity of the task and thus allows “intuitive” navigation of large combinatorial spaces, as in chess:

“Intuition is the ability to assess a situation, and without reasoning or logical analysis, immediately take the correct action. An intuitive decision can arise either as the result of long thought about the answer to the question, or without it”

*—(Beim, 2012, p. 10)*.

The experience of “intuition” is produced by the ability to relate, via sufficiently tight coordinations, particular moves to the global objective (winning the game)—a move is “sensed” to improve or degrade the overall position (in the chess literature, this ability has been compared to a GPS in the player’s mind showing whether moves take one towards or away from the goal (Palatnik and Khodarkovsky, 2014)). Such guiding intuition is not confined to chess but is a universal attribute of complex analysis and problem solving that is informed by coherent models.

“The mass of insufficiently connected experimental data was overwhelming…however, I soon learned to scent out that which was able to lead to fundamentals and to turn aside from everything else, from the multitude of things which clutter the mind and divert it from the essential”

*—(Einstein, 1949, p. 17)*.

**Figure 7:**
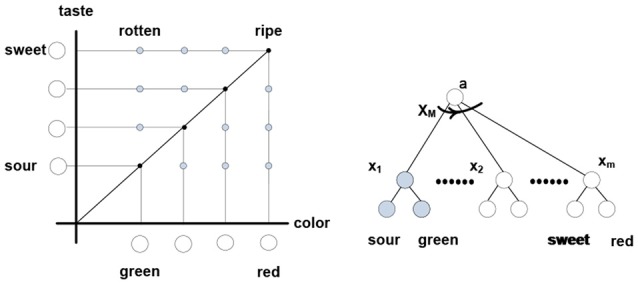
**Thinking “apple is ripening” involves rotating “apple” packet vector from the (sour-green) to (sweet-red) terminal positions via some intermediate angular positions.** Neuron *x*_1_ responds to co-firing of “sour” and “green” neurons, *x*_M_ responds to co-firing of “red” and “sweet,” etc. Neuron *x*_M_ responds to the firing succession *x*_1_ → *x*_2_ → … →*x*_m_ formed of diagonal elements in the color-taste matrix. Thinking “apple is rotting” engages different elements residing in different rotation trajectories. In a simulation, firings can be associated with different values, contracting the matrix attributes and assigning value to the “ripening” trajectory; namely, the sum of values in the *x*_1_ → *x*_2_ → … →*x*_m_ firing succession (spur of the matrix).

Navigating and connecting massive sensory data requires a model that guides subsequent probes and enables determination (however approximate) of whether the data lies within the range of variation afforded by the model, or falls outside the range and invalidates the model. As per Figure 1, probabilistic prediction and inference are at the foundation of the modeling process.
(c) Coordinations in systems of nested packets can be expressed as optimization operations in vector spaces (Dorny, 1975) and as functions over tensors or multi-vectors (Clifford vectors) of geometric algebra (Hestenes and Sobczyk, 1999; Doran and Lasenby, 2003). Complexity reduction in such systems can involve rank reduction and tensor contractions.
(d) In the nervous system, complexity reduction can involve neurons responding to trajectories of packet vectors; that is, particular successions of their angular positions. In other words, such neurons respond to particular thinking patterns, as illustrated in Figure 7.

### Summary

This section outlined a parsimonious theory of understanding where foundational ideas in systems neuroscience (Hebbian assembly) and probabilistic learning theory (variational free energy minimization) converge on the notion of a neuronal packet—a neuronal assembly “wrapped” in Markov blanket. Cognitive processes are defined as operations on neuronal packets providing a unifying formalism to express the function of understanding as well as phylogenetic and ontogenetic development of intelligence culminating in that function: allocating neurons—allocating cohesive neuronal groupings—adjusting groupings—apprehending coordinated adjustments—combining and coordinating groups (*mental modeling*). Psychologically, the process encompasses the progression from sensing, to perceiving, to understanding. Mathematically, this formalism suggests operations on vector spaces (via a geometric calculus).

The ensuing theory grounds cognitive development in thermodynamics, suggesting a straightforward relationship between self-organization and evolution (packets are thermodynamically sculpted and operations evolve). Evolution engages an interplay between the internal (packet manipulations demand energy) and external processes (where the environment supplies energy), propelled by the need to improve energy efficiency. Organism-environment coupling is probabilistic, allowing a dual account: doing work to extract energy manifests as sampling and information gathering. The energy-saving tendency to maintain cohesive and stable packets is motivated by the minimization of surface tension in the packet boundary surface. Surface tension is a fundamental parameter expressing the thermodynamically favored direction of internal processes in any system. In a neuronal system, favored processes include increasing cohesion (reducing interface area in individual packets) and merging (reducing the total interface across the packet set). Minimization of surface tension entails minimization of a thermodynamic free energy in packet surfaces and equates to avoiding surprise (minimizing variational free energy in probabilistic inference). On that theory, packets are the substrate of inference.

One might ask whether the solutions that minimize variational free energy are stable and—from a technical perspective—are these functionals convex. By virtue of the dynamic and itinerant nature of biological systems (especially in the context of a circular causality implicit in self organization), it is highly unlikely that the energy functionals describing behavior are convex. Heuristically, this means that there will be many minima—or solutions. The implicit multi-stability provides a nice mathematical image of speciation—and indeed variants within any phenotype. In other words, there is no unique free energy minimum, in the same sense that there is no unique phenotype; each system adapts to its own econiche—finding its own solution.

The notion that quasi-stable neuronal packets—and their manipulation—underlie perception resonates with theories that associate perceptual units with quasi-stable solutions in mean field models; for example, neural field models that account for the neurogeometry of the cortex and the impact of visual input (e.g., Sarti and Citti, 2015). According to Sarti and Citti (2015), in the absence of visual input, quasi-stable solutions correspond to hallucinatory patterns. Notwithstanding the possibility of quasi-stable neuronal clusters engendering hallucinatory experiences, our theory predicates mental modeling on the formation of quasi-stable packets that maintain their integrity throughout episodes of absent and/or varying input. Such quasi-stable units allow the experience of continuing, self-identical objects that arise from (i.e., are superposed upon) discontinuous and varying sensory streams. More generally, the neuronal packet model is compatible with the mean field models that furnish a dynamics of neuronal systems from metastability and symmetry breaking—and associating system behavior under stimulation with quasi-stable states and active transient responses (Wilson and Cowan, 1972, 1973; Bressloff et al., 2002). Examining conceptual commonalities and reconciling differences between these models may help overcome their inherent limitations (e.g., Destexhe and Sejnowski, 2009) and offer synthetic perspectives.

## Analysis

This section compares the proposal in the preceding section to other theories described in “Theories of Understanding”. Since our proposal rests on the notion of neuronal packets, we discuss how the idea conforms to the principles of neuroscience and present some recent data concerning the properties of neuronal structures consistent with those attributed to neuronal packets. Finally, we consider an approaches to understanding motivated by complementary ideas based on “intuitive physics engines”.

### Comparing Theories

The theories in “Theories of Understanding” complement our formulation. Moreover, they appear to reflect different facets of understanding, as conceptualized above. The “foundational theory of understanding” (Newton, 1996), which grounds understanding in self-directed (volitional, attentive) activities reconciling sensory inflows with memory structures and current goals, is consistent with our theory that associates understanding with goal satisfaction via self-directed allocation of neuronal resources. The idea that evolution has gradually shifted response targets away from the sensory periphery, producing internal efferent-afferent loops that can be decoupled from the motor output (Humphrey, 2000, 2006) is formally expressed in the model of self-adaptive resource allocation.

The key insights in the theory of understanding by Chart (2000) appear to be formally expressed and substantiated by our treatment. Chart (2000) derives understanding from simulations involving effortful (work- consuming) operations on mental models built of “mutors”:

“Mutors are both the building blocks and the* motors* of mental models…mutors are active: they *actually do the work* on the input, and produce the output. They are not rules by which the input can be transformed into the output; rather, they are machines which effect the transformation”

*—(Chart, 2000, p. 47)*.

These intuitive notions correlate closely with the idea of effortful vector rotation and other ideas (see Figure 6; note similarities between Chart’s theory and Shannon’s DA. The theories also differ in that one is centered on the work requirement and the other is oblivious to it).

The *doing work* requirement in Kauffman (2000), predicating intelligence on the ability to invest energy in performing thermodynamic work cycles directed, in part, on erecting constraints for the subsequent energy releases, appears to be fully upheld in our theory (e.g., boundary energy barriers constrain composition and movement of packet vectors thus constraining energy release in vector rotation which, in turn, constrains condition at the boundary). The idea of associating intelligence with “approximate constrained optimization” in the service of need satisfaction (Glasser, 1984; Werbos, 1996, 1998) is inherent in the notion of probabilistic resource optimization. Our proposal ascertains a reciprocal and complementary relationship between probabilistic resource optimization via resource grouping, statistical explanation (Salmon, 1970) and probabilistic inference, as discussed above.

Simplification (Kitcher, 1981) and compression—postulated to be the definitive characteristic of explanation (“comprehension is compression”, Chaitin, 2006)—are the product of enfolding, collapsing multiple resources into a single unit. In essence, alternating enfolding–unfolding serve to break large combinatorial problems into sets of much smaller ones, yielding profound complexity reduction. Furthermore, simplification is isomorphic with complexity minimization inherent in minimizing variational free energy and, by implication, thermodynamic complexity costs.

Finally, our theory gives operational expression to some of the central claims in the psychological theory of understanding. Developmental psychology predicates development of a capacity to understand, from infancy to maturity, on the growing ability to conduct “co-instantaneous mental coordinations” and thus apprehend relations abstracted from the current sensory input:

“…to coordinate data yielded by his own actions the child must appeal to unobservable, deductive relations which transcend his actions”

*—(Piaget, 1978, p. 12)*.

Our proposal defines processes underlying “mental coordinations” and makes them responsible for all levels of understanding, from handling toys to formulating abstract theories. From the resource optimization standpoint, coordinating packets in nested packet groupings provides a scalable mechanism for compression and complexity reduction. From the psychological standpoint, coordination combines disparate and unrelated entities into “situations” imbued with meaning. That is, meaning is imputed by relations.

### Neuronal Packets

A “neuronal packet” is a system-theoretic idea derived from conceptualizing the nervous system as a probabilistic resource optimization system with self-adaptive capabilities (Yufik, 1998b). The starting point was attempting to formulate Hebbian assemblies (Hebb, 1949, 1980) as material entities: what makes assemblies distinct, how does the system “know” where one assembly ends and another begins? Once formed, why wouldn’t assemblies succumb to entropic erosion and dissolve momentarily? Drawing on Haken (1983, 1993), packets were hypothesized to be formed by phase transitions in associative networks and sculpted by an interplay between thermodynamic forces (reduction of thermal free energy in the inter-phase surface) favoring coalescence and forces of lateral inhibition resisting coalescence. This interplay dynamically optimizes responses: through lateral inhibition, packets capture regularities in the sensory stream.

Arguably, the existence of boundary mechanisms was implicit in the notion of assembly, the consequences (structure variation, induction of meaning, etc.) were fully anticipated by Hebb:

“…we have come to a classical problem…the meaning of “meaning”.… a concept is not unitary. Its contents may vary from one time to another, except for a central core whose activity may dominate in arousing the system as a whole. To this dominant core, in man, a verbal tag can be attached; but the tag is not essential. The concept can function without it, and when there is a tag it may be only a part of the “fringe”. The conceptual activity that can be aroused with a limited stimulation must have its organizing core, but it may also have a fringe content, or meaning, that varies with the circumstances of arousal”

*—(Hebb, 1949, p. 133; see Figure 5)*.

The notion of *intrinsic organization* of cortical activity “that is so called because it is opposed to the organization imposed by sensory events” (p. 121), the necessity for assemblies to be sustained over time (p. 121), the possibility of forming “latent” associations between stimuli that have never co-occurred in the past (p. 132), the “coalescence” of assemblies (p. 132), and numerous other ideas in Hebb (1949) place the packet concept within Hebb’s framework.

The concept of a “neuronal packet” is consistent with other system-level theories of cognition. The theory of neuronal group selection (TNGS; Edelman, 1992, 1993; Edelman and Tononi, 2000) associates cognitive functions with the formation of “neuronal groups” and establishment of “re-entrant mappings” between groups (Edelman and Gally, 2013; see Figure 6). In Gestalt psychology, packets manifest in the notion of “gestalt bubbles” (Lehar, 2003a,b), or “segregated wholes” that enable meaning (“… meaning follows the lines drawn by natural organization; it enters into segregated wholes” (Köhler, 1947, p. 82)). Significantly, “segregated wholes” were subject to forceful manipulation (the idea organizing “force fields” in the brain that “extend from the processes corresponding to the self to those corresponding to the object” (Köhler, 1947, p. 177; 1948)). The idea of “forceful” interactions was later associated with the activity of consciousness: in the brain, consciousness is “put to work” exerting a controlling influence on the stimuli-triggered and volitional (self-generated) motor responses (Sperry, 1969). Interestingly, the notion of force fields as underlying perception has been revisited in the context of gauge theories for the brain using variational free energy as the underlying Lagrangian (Sengupta et al., 2016). Formally, this is closely related to the autopoietic destruction of (free energy) gradients in synergetic formulations of brain function (Tschacher and Haken, 2007).

A “neuronal packet” is a speculative concept—the implicit packets (or assemblies) are not amenable to direct observation but have to be inferred in terms of their functional connectivity and underlying conditional independence. However, recent empirical data appears to uphold the concept. Packets are thermodynamically plausible because their [re]use minimizes energy expenditure. That is, the possibility of re-use is inherent in the packet idea. Reusable neuronal groups (“bubbles”) were discovered in the hippocampus of awake, free-moving animals (mice; Lin et al., 2005, 2006; Tsien, 2007). Empirical verification was enabled by recent technical advances allowing simultaneous recording of activity of 260 neurons: recordings were made in the CA1 region in animals subjected to different perturbations (shaking, elevator drops, air puffs) and in the resting state. Multiple discriminant analysis (MDA) was carried out over half-second sliding windows in recordings accumulated over several hours, revealing the formation of distinct “bubbles”, Or groupings of neuronal activity that were well separated in the functional 3-D space (contracted by MDA from the 520-D space). The ensuing bubbles represented “integrated information about perceptual, emotional and factual aspects of the events” (Tsien, 2007, p. 55). After the “bubbles” were formed, subsequent responses could be characterized in different compositions, e.g., an “earthquake” type situation begins in the “resting bubble”, transits to the “earthquake bubble” and returns to the “resting bubble”—thus following a distinct trajectory in the functional space.

The possibility of resource tuning (changing resource characteristics depending on those of the task) is inherent in the concept of resource allocation (see Figure 2). Task-dependent changes in the receptive fields of individual neurons (see rotation of neuronal response vectors) have been demonstrated in a broad range of tasks and conditions including different stimulation modalities (auditory, visual) and durations of exposure (Fritz et al., 2003, 2007; Kohn and Movshon, 2004; Elhilali et al., 2007). For example, recordings of individual neurons in A1 in ferrets performing tone-discrimination tasks revealed distinct and predictable changes in spectro-temporal receptive fields (“task-specific signatures”; Fritz et al., 2007). In the earlier experiments, neurons in the prestriate area V4—in monkeys attending to visual stimuli—demonstrated robust attentional gating of their receptive fields: a neuron having two stimuli within its receptive field selectively suppressed its responses to one or the other stimulus depending on the task (Moran and Desimone, 1985).

Task-dependent changes in the responses of neuronal populations (rotation of population, or packet vectors) were demonstrated by Georgopoulos and his group in studies of neuronal correlates of target reaching in monkeys. Neurons in M1 are broadly tuned to the direction of movement, with each neuron exhibiting a preferred direction—defining the orientation and the magnitude of the neuronal response vector. It was shown that population response vectors—obtained as the vector sum of weighted neuronal response vectors over the population of responding motor neurons—track the direction of the hand movement (Georgopoulos et al., 1988, 1993). In a similar fashion, weighted sums of neuronal responses over populations of sensory neurons were shown to align closely with the overt characteristics of sensory processing (Jazayeri and Movshon, 2006). Furthermore, it was shown recently that population responses adapt to task variations, involving subsets of neurons particularly relevant to the current task (“high-precision neurons”; Purushotaman and Bradley, 2005).

The overall approach of conceptualizing cognitive processes as optimization of neuronal resources has received experimental support and theoretical emphasis in the recent studies of visual perception (Gepshtein et al., 2013) and the analysis of candidate mechanisms in the brain capable of anticipation and long-term planning (“prospective optimization”; Sejnowski et al., 2014). Perhaps, the most compelling argument in favor of the present theory can be garnered from the work reported by Ito (1993, 2008), Salman (2002), Baillieux et al. (2008); Ellis and Newton (2010), Murdoch (2010), and Rosenbloom et al. (2012) suggesting a possibility that mental activities are controlled by internal models in the cerebellum (Ito, 2008), with movement and thought engaging identical control mechanisms (Ito, 1993). On the theory that understanding boils down to packet coordination, pieces of the understanding puzzle seem to be falling in place. That is, the critical function of packet coordination hypothesized in Figure 6 may be evident in the cerebellum.

Key components of “understanding” include value-assignment (reward likelihood attribution), packet mobilization and effortful, context-sensitive variation, packet coordination, output suppression and response selection. These components map, under a gross simplification, onto a functional neuroanatomy comprising prefrontal cortex (PC), subcortical structures; including the basal ganglia, thalamus, and cerebellum, and the limbic system (Rosenbloom et al., 2012). The orbitofrontal, anterior cingulate and dorsolateral regions in PC interact with each other and the limbic system and subcortical structures. In particular, the orbitofrontal cortex and limbic system participate in reward-attribution, while the dorsolateral and anterior cingulate regions “facilitate intellectually effortful decisions” (Rosenbloom et al., 2012, p. 256). Frontal areas are involved in response suppression, while the cerebellum mediates a key mechanism of understanding: packet coordination. Via the cerebellum, precise timing—necessary for sensorimotor coordination (Salman, 2002)—becomes an integral part of situational understanding that is manifest in the ability to not only compose, in the mind, coordinated activities fine-tuned to the current situation but also to identify proper moments for releasing and terminating them.

Energy barriers play a crucial role in coordinated timing. On the present theory, folding into packets creates a continuous energy landscape in associative networks (peaks and valleys form energy barriers that separate pools of neurons endowing them with a conditional independence that create Markov blankets). The implicit barriers may be regulated by the limbic system (regulation of the “cortical tone” (Luria, 1973)), via the classical ascending neuromodulatory systems. For example, down regulation (stress, fear, low motivation) raises energy barriers, while up regulation (joy, arousal, high motivation) lowers them. This sort of regulation or (neuromodulatory) arousal, directly affects cognitive performance as follows. Optimal performance requires optimal “cortical tone” (underlying the Yerkes—Dodson law of optimal performance (Eysenck and Keane, 1995)). Excessive down regulation blocks attentive access to packet internals (as in suddenly forgetting a familiar name) or arrests attention within a packet (vacillation, inability to escape from recurring thoughts). By contrast, excessive up regulation precludes sustained focus and predisposes to spurious associations. In pathological extremes, the landscape is either flattened, turning sensory inflow into undifferentiated flux (e.g., Alzheimer’s disease), or loses integrity and decomposes into pockets of narrowly constrained skills (e.g., autism). When a packet dissolves, the contents are not forgotten but irrevocably lost. We shall re-visit this point briefly in the discussion.

The mechanism of mental modeling is ubiquitous across species. For example, sensing a prey initiates hunting behavior in a snake. If the prey suddenly disappears, the snake starts searching for it but only in the vicinity of the location where the prey was last sensed. By contrast, a dog chasing a prey that goes out of sight (e.g., a rabbit disappearing behind bushes) can initiate an interception maneuver; i.e., running towards a location where the prey is likely to re-emerge (Sjölander, 1995). Figuratively, the snake’s hunting model contains one packet whose boundaries are statistically determined and genetically fixed (the radius and duration of search are consistent with the behavior of animals typically consumed by snakes—thus yielding adaptive fitness). Dogs and other higher animals possess repertoires of specialized packets amenable to situation-sensitive variations (a prey’s velocity, distances, etc.). Chimpanzees can combine some genetically available activities (reaching with a stick, piling up objects and climbing to obtain a reward reflect their genetic repertoire) but coordinating such activities appears to be approaching the limits afforded by their nervous system. Human modeling capabilities in infancy are rudimentary (e.g., at 6 months, infants search for a toy after it was covered but, if the toy is removed and placed (in full view) under a different cover, they keep searching for it where it was first perceived (Bower, 1974)). Human capabilities develop rapidly, from coordinating a few variables in handling toys (e.g., ball placement in a toy catapult, given the distance to the target) to coordinating deeply nested variable structures in the creation of abstract theories. We propose that the formalism of neuronal packets and packet coordination characterizes essential features of the underlying mechanism at all stages of cognitive development.

So far, understanding and mental modeling have been discussed in the context of problem solving and prediction (Toulmin, 1961), without addressing the impact of emotion on these cognitive activities. The thermodynamic framework suggests a natural expression of that impact (Yufik, 1998a), by identifying emotional control with thermoregulation and temperature with the level of arousal (it is interesting to note that Aristotle attributed to the brain the function of thermoregulation, Gross (1995)). In particular, the neuronal packet model represents boundary free energy (the height of packet energy barrier) U as a function of temperature approximated as U(T) = σ – Tdσ/dT where σ is a stability coefficient computed as the ratio of the summary strength of the internal vs. external associative links in the packet (σ > 1: such that the packet disintegrates when σ approaches unity, bringing U(T) in to the vicinity of kT, where k is the Boltzmann constant). Increasing T lowers the barriers while decreasing T (stress, fear, anxiety) results in their elevation. Low barriers enable easy (low energy cost) transitions between packets (expansive, compositional thinking) while elevated barriers hamper the transitions.

Temperature variations can be local (focused thinking) or global (diffuse). Diffuse temperature increases lower energy barriers and “shake up” the system, entailing re-distribution of neurons among packets, followed by focused (selective) manipulations in the resulting structures (the term “cognition” derives from the Latin “cogito” meaning “to shake together”, “intelligence” derives from the Latin “intelligo” meaning “to select among”, Koestler, 1964, p. 120). As noted earlier, the overall temperature dependency of the packet system approximates the Yerkes-Dodson law of performance (optimal levels of arousal yield optimal cognitive performance). More generally, temperature regulation engages global self-regulatory loops allowing the organism to reconcile conditions in the outside with those inside and thus maintain a form of homeostasis. Arguably, thermal regulation transcends the hierarchy of functional levels in the organism—from changes in the cell membrane permeability and neurotransmitter flow (e.g., changes in the release, reuptake and repriming of synaptic vesicles; the micro level) to changes in packet composition (the mesa level), and further to emotional shifts entailing changes in overt macro responses (advance or retreat; the macro level). These views are generally consistent with those formulated in Damasio and Carvalho (2013) and Damasio and Damasio (2016).

### Alternative Theories

A recent theory of cognitive mechanisms involved in the understanding of physical scenes (e.g., a determining whether a stack of blocks is going to hold or to topple) derives understanding from the operation of an “intuitive physics engine” (IPE) combining simulation of interaction between objects with probabilistic inference, by treating simulation runs as statistical samples (Battaglia et al., 2013). Simulating interactions is the crux of the matter, how is this accomplished in IPE? To demonstrate human-like performance, IPE employs open dynamics engine (ODE^1^) offering a library of routines (equations, methods and algorithms) to simulate rigid body dynamics. If IPE succeeds in emulating humans, what would this tell about the mechanisms of scene understanding in the brain? Stated differently, what makes IPE brain-like?

Three constraints in employing the ODE library are claimed to qualify IPE as a theory of scene understanding: only elementary rules of physics are selected in ODE, Monte Carlo procedures inject probabilities into simulation runs, and inference calculations are carried out to a crude approximation. Consider applying these constraints in a toy catapult problem (e.g., balancing two objects on a plank): w_1_L_1_ = w_2_L_2_ is the most elementary rule, simulation varies the values of L_1_ and L_2_, probability distributions are associated with variation ranges L_1_ and L_2_, and all calculations discard small terms and round the results. If that is what underlies understanding, the question remains: how is the rule w_1_L_1_ = w_2_L_2_ obtained, represented and exercised? The probabilistic inference and approximation components in IPE only postpone the inescapable conclusion that understanding boils down, literally, to mental arithmetic. With that, any human-like behavior can be readily imitated and explained (e.g., a child failing to understand that ball needs to be moved away from the center of the catapult when the distance to the target increases, has her Monte Carlo flip the sign, i.e., computes L_2_ − ΔL_2_, instead of L_2_ + ΔL_2_,).

In short, results in Battaglia et al. (2013) appear to demonstrate that combining methods of analytical mechanics with probabilistic inference allows rough and quick assessment of interaction dynamics in simple mechanical systems. Whether these results have anything to do with human understanding or intuition is open for debate. In lieu of entering the debate, this article has outlined a complementary approach to the issue.

## Discussion and Suggestions for Further Research

Brain is complex, dynamic self-organizing system (Bressler, 1994; Singer, 2009). Self-organization requires a flow of thermodynamic energy through a system acting as a conduit between an energy source and energy sink. At equilibrium, energy transfer by thermodynamic forces is accompanied by generation of entropy. Deviations from equilibrium is accompanied by a decrease in the rate of entropy production, eventually producing conditions where stable structures emerge in the form of spatial (e.g., Bérnard cells), temporal (e.g., Belousov-Zhabotinsky reaction) or spatiotemporal structures (Glansdorff and Prigogine, 1971; Prigogine and Stengers, 1984, 1997; Prigogine, 1994; Bak, 1996; Jensen, 1998). The brain belongs in the continuum of self-organizing systems (Bressler, 1994; Kelso, 1995; Camazine et al., 2001). Sustained self-organization in far-from-equilibrium systems is contingent on the existence of internal mechanisms capable of removing entropy from the volume occupied by the system and depositing it outside the volume (Morowitz, 1978, 1979; England, 2013; Prokopenko et al., 2014). The development of intelligence implies a reduction of entropy within the brain’s volume—to levels allowing emergence of stable structures that can both amplify energy inflows and direct the investment of a growing portion of that inflow towards creating more entropy reducing structure. In a sense, a self-organizing (self-adaptive) system keeps folding upon itself, producing increasing degrees of internal order. Human intelligence requires a degree of order, engendering stable but flexible structures (neuronal packets) and reproducible internal processes (thinking). This combination gives rise to the experience of interacting with an orderly environment amenable to understanding, as follows.

The requirements of facilitating energy import from the outside—and structure generation of the inside—converge when structures are flexible (but stable) and reflect regularities in the external conditions. With that, reciprocity is established between internal “objects” and environment. A self-organizing system becomes aware of the “objects”— including itself as an object; i.e., when objects become amenable to internal manipulation, establishing relations between objects expressing higher-order regularities in the environment. The availability of such manipulations rests on having reduced the rate of entropy production, down to levels that allowing reversibility of thinking. That is, no thinking is possible if one cannot: (1) dwell on object A; (2) switch from object A to object B and return to B; and (3) keep all the objects intact in the course of 1 and 2. Reversibility endows quasi-stable objects with self-identity, thus rendering thought possible and making the environment (the universe of persevering, self-identical objects) understandable. The relationship between reversibility and understanding is manifest in the foundational principles of psychology, logic and mathematics.

In psychology, this relationship was first articulated in the last century by Piaget, in the form of a reversibility principle and the notion that cognitive structures—and operations on those structures—in mature adults acquire the property of algebraic groups. In logic, the relation underlies The Law of Identity formulated by Aristotle as the key axiom from which reasoning derives. The Law of Identity (**A** ≡ **A**) (and the corollary of non-contradiction and excluded middle) asserts preservation of self-identity in things despite changes. Things neither appear nor disappear spuriously, they remain self-identical over time and do not change without a cause. Finally, in mathematics, the relation is expressed in the foundational principle of set induction and cardinality attribution formulated by Cantor (1915/1955):

“We will call by the name “power” or “cardinal number” of *M* the general concept which, by means of our active faculty of thought, arises from the aggregate *M* when we make abstraction from *m* and the order in which they are given”

*—(Cantor, 1915/1955, see Tiles, 1989, p. 99)*.

In short, set is induced on a group by the “active faculty of thought” that treats the group, reversibly and alternatively, either as a manifold or as a unit abstracted from the manifold.

The criteria of causality are hard to explicate (e.g., leading to the recent notion of “graded causation” (Fitelson and Hitchcock, 2011; Halpern and Hitchcock, 2015)) but, nuances aside, causality concerns a relation between some A and B: changes in A are (or are not) the cause of changes in (B). By contrast, the set operation dwells on A. The operation underlies mathematics (and abstractive thinking in general) and enables compositionality; i.e., combining A and B into a new unit A, B → (AB) amenable to reversible decomposition (AB) → A, B, and so on, indefinitely.

According to the theory of neuronal packets, the above principles are rooted in (and express) packet unfolding/enfolding and inter-packet coordination (causality). Unfolding gives access to the packet’s sensory contents, while enfolding abstracts from them. Alternating between enfolding and unfolding can be visualized as moving up and down a cone; with the sensory data at the base. On the way up, the sensory component is reduced—and is completely removed (abstracted away) at the apex. Symbolic labels that could be attached at the apex (e.g., labels “apple” and “Apple computer”) have no sensory overlaps with the corresponding objects. The essence of thinking is effortful packet manipulation, with the process alternating sporadically between imagining and reasoning (syntactic manipulation of labels). Crucially, the process is different from—and does not reduce to—*pattern recognition*. This contention will be discussed elsewhere.

The development of order in self-organizing systems implies the emergence of Markov blankets; i.e., encountering a confluence of conditions that allows the system to self-segregate, or fold into components that remain coupled to the system but acquire conditional independence. In living organisms, mechanisms start to form that regulate the “permeability” of the blankets, i.e., facilitating inflow of energy and matter necessary for sustaining independence and integrity at the level consistent with survival. One might imagine that further development creates higher-order regulatory mechanisms comprised of nested components “wrapped” in Markov blankets.

When analyzing the thermodynamic underpinnings of life, Schrodinger introduced the notion of negentropy extraction: “the device by which an organism maintains itself at a fairly high level of orderliness (low level of entropy) really consists in continually sucking orderliness from its environment” (Schrodinger, 2006, p. 73). Negentropy extraction involves active sampling and harvesting of information from the environment. The induction of Markov blankets and increase of order via partitioning of associative networks into nearly homogeneous subsets (neuronal packets) equates to internal generation of information (Salmon, 1970). Thermodynamic free energy is therefore diverted from dissipating organismal structure and is stored in ATP molecules at the packet surface, to be released in the work of composing and re-shaping packets for further free energy minimizing inference. Our theory defines the increase of order via constructing models as negentropy generation (orderliness is manufactured inside the system).

Minimization of boundary free energy can drive self-organization and self-assembly in microstructures (Syms et al., 2003) and influence first-order phase transitions, inducing critical phenomena (surface-induced order and disorder (Lipowsky, 1984)). The coexistences of phases in a first-order transition is described by Landau-Lifsitz potential with several minima, with spontaneous symmetry breaking (e.g., packet formation) on obtaining one of the minima (producing order and the disordered phase characterized by a vanishing order parameter (Lipowsky, 1984)). In general, identifying the thermodynamic variable with the surface area of a packet offers a hypothetical Lagrangian or Lyapunov function that poses some interesting analytic and practical questions. From a technical point of view, it motivates a formal analysis of the relationship between the surface area (thermodynamic free energy) and variational free energy. From a practical point of view, the surface area can be treated as an order parameter, which is either minimized or conserved—in accord with Hamilton’s principle of stationary or least action.

Transition from negentropy extraction to negentropy generation encompasses a continuum of intelligent processes, from rudimentary (plant intelligence, e.g., Trevawas, 2002; Marder, 2013) to the most elaborate (human intelligence). In the latter, a spectrum of mechanisms can be involved operating in conjunction with neuronal mechanisms; e.g., from limbic neuromodulation to glial cell function (Chung et al., 2015); from synaptic processes to microtubules (Penrose, 1997). All such mechanisms exploit thermodynamic forces to optimize energy extraction and utilization in the interest of survival (e.g., sunflowers tracking the sun). Accordingly, the formalism of self-adaptive resource optimization applies across the continuum of biological intelligence. Emulating biological intelligence in artifacts would require a range of designs, including analog (super-Turing network (Siegelmann, 1999; Cabessa and Siegelmann, 2011)), digital and digital-analog hybrids.

Our proposal associates self-organization in the physical substrate with minimization of free energy, and asserts isomorphism between variational and thermodynamic expressions of free energy. Under both expressions, the process involves self-partitioning in the substrate yielding internally cohesive and externally weakly coupled (statistically quasi-independent) components. As astutely noted by a reviewer, the concept of energy minimization resonates with some classical techniques in pattern analysis (e.g., energy minimization in Hopfield networks) and image processing. In general, minimization of an “energy functional” is used to obtain image segmentation into “meaningful” regions (“objects”) having uniform feature intensity and separated by non-uniform, low-intensity patches. Minimization can be sought of some local energy-like expression (Lucas and Kanade, 1981) or a global energy functional (Horn and Schunck, 1981; Bruhn et al., 2005). In the former case, the “energy functional” takes the form E(u, B) → min where u is the smoothed image and B is a curve segmenting the image (i.e., the union of “object” boundaries; Mumford and Shah, 1989; Shah, 1992).

Mathematical ideas motivating boundary detection by minimizing energy functionals (Mumford and Shah, 1985) appear to be converging on our proposal postulating free energy minimization in the interface or boundary separating neuronal packets from the surrounding structure, thus providing further support to the hypothesis that packets underlie perception of “objects.” Note that our overall proposal deals with models of input (rather than percepts) and thus calls for expanding the conceptual basis and the corresponding mathematical apparatus, as compared to those employed in image processing. In particular, the free energy minimization requirement is associated not only with segmenting images into packets (“objects”) but, crucially, with the subsequent operations on packets, such as coordinated rotation of packet vectors. In other words, the energy functional needs to be extended to include minimization over two variables: the boundary energy and the action. We believe that examining relations between energy-like function minimization in image processing and variational and thermodynamic energy minimization in mental modeling is likely to yield informative and practically useful results, presenting a challenge for further research.

It is interesting to note that the vector manipulation formalism adopted in the present theory overlaps, to a degree, with the theory of morphogenesis in Thom (1975). In particular, the theory expresses morphogenesis (change of form) in a system M in terms of a vector field X on M determining the system’s macroscopic dynamics. However, the overlap is limited since the intent was to “construct an abstract, purely geometrical theory, independent of the substrate of forms and the nature of the forces that create them” (Thom, 1975, p. 8). Similar attempts can be found in other system-theoretic studies of complex structures (e.g., Casti, 1979). Most system theories, including Thom (1975), focus on the general conditions of stability and resilience; i.e., the system’s ability to absorb external disturbances without dramatic consequences for its steady-state and transient behavior. By contrast with system-theoretic proposals, the present proposal resonates with the objective reinstating the primacy of action and bodily grounded experiences in the theory of intelligence (Nunez and Freeman, 2014) and is interested in the physical properties of the substrate and the forces, seeking to relate them to resilience and adaptive changes. Nonetheless, system theories offer a rich mathematical apparatus and key insights (e.g., concerning the role of topological factors in biological morphogenesis (Thom, 1975)), that may contribute to a comprehensive theory of cognition.

### Summary

Life emerges in networks of interacting material entities under a confluence of conditions that allow regions in the network to fold into bounded units statistically independent from the environment. Sustaining life requires regulating the flow of energy and matter through the boundary. The dual requirement of maintaining independence from the environment, while extracting sustenance from it, is resolved in progressively improving regulatory mechanisms ascending from the boundary to the internals. The progress is enabled by folding in neuronal networks and culminates in mental modeling involving manipulation of folded units (packets).

A detailed examination of the above hypothesis suggests a metaphor of brain function that comprises Bayesian and Aristotelian components, as follows. The interaction between an organism and its environment is probabilistic (no action is guaranteed to yield the expected outcome), necessitating Bayesian inference to predict and prepare for counterfactual outcomes before their onset; i.e., the cybernetic or Bayesian brain (Conant and Ashby, 1970; Knill and Pouget, 2004; Seth, 2014). Self-organization creates structures and operations in the system allowing logical inference; i.e., the Aristotelian brain. The Aristotelian brain builds on the foundation of the Bayesian brain in the course of self-adaptive resource optimization. The need to invest work in operating on structures equilibrates the Aristotelian-Bayesian system in the brain: self-partitioning into packets establishes both reference sets for Bayesian inference and a trade-off between the amount of cognitive work the system can invest and the amount of surprise it can tolerate.

The self-adaptive resource optimization framework (Yufik, 1998b, 2002; Yufik and Malhotra, 1999; Yufik and Sheridan, 2002) offers a simple account of cognitive processes, highlighting the crucial role of Markov blanket induction in neuronal systems, as a pivotal optimization mechanism.

From the perspective of Bayesian inference, induction equates to dynamic partitioning of large inference problems into a hierarchical succession of simpler problems, minimizing complexity (through dimension reduction) with the least loss of accuracy. Anticipatory inference (e.g., counterfactual prediction) is integral to optimization. This formalism is consistent with the functional organization of memory, distinguishing long-term (model parameters) and short-term (postdictive) components: in this (Bayesian) setting structure learning and inference can be expressed as optimization on vector constructs, such as Clifford vectors or tensors (e.g., Dorny, 1975; Smolensky, 1990; Doran and Lasenby, 2003).

From the perspective of physics, abductive reasoning equates to placing associative networks into regulated variational free energy landscapes where cohesive subnetworks (“bubbles”) reside in valleys separated by energy barriers. This (variational and thermodynamic free) energy landscape defines expenditures (energy consumption and dissipation) in terms of the computational complexity—accuracy trade-offs and motivates optimization (Sengupta et al., 2013). From the perspective of psychology, induction underlies the unparalleled efficacy of human reasoning, by enabling transition from sensation to perception and from perception to understanding.

From the perspective of systems neuroscience, the function of understanding appears to be mediated by the Aristotelian-Bayesian brain via collaborative engagement of the thalamo-cortical system (associative network), the limbic systems (emotive thermoregulation) and the cerebellum (coordination). The theoretical perspective offered in this article is based on a fundamental, cornerstone of systems neuroscience (Hebbian assembly), by attributing biophysical properties to the assemblies that, arguably, are implicit in—and have been anticipated by—the original concept.

Finally, from the perspective of technology, implementation of the optimization and induction mechanisms speaks to a transition from machine learning to machine understanding. Advances in machine intelligence over the last half century have been associated primarily with perfecting techniques for computing weight distributions in fixed topology (perceptron-type) networks yielding a mapping between the input and output vectors (learning, pattern recognition). The store of algebraic ideas that have been employed in the task is rich, going back to Tichonov’s regularization and iterative error reduction methods by Gauss, but finite and appearing, despite the recent strides (e.g., deep learning), to be nearing exhaustion. Simulation of understanding involves networks of varying topology and operations on dynamic vector structures, with the weights intact. Implementing such simulations could exploit algebraic ideas that have been largely untapped, promising advances in autonomous systems and other critical applications that, arguably, are not accessible via the methods of machine learning. These distinct but complementary perspectives indicate possible avenues for further investigation.

## Author Contributions

Both authors collaborated in writing the article. All authors listed have made substantial, direct and intellectual contribution to the work, and approved it for publication.

## Conflict of Interest Statement

The authors declare that the research was conducted in the absence of any commercial or financial relationships that could be construed as a potential conflict of interest.

## Footnotes

^1^http://www.ode.org
